# Apoptosis Inducing Effect of Plumbagin on Colonic Cancer Cells Depends on Expression of COX-2

**DOI:** 10.1371/journal.pone.0018695

**Published:** 2011-04-29

**Authors:** Bharathi Raja Subramaniya, Gayathri Srinivasan, Sakeena Sadullah Mohammed Sadullah, Nimitha Davis, Lakshmi Baddi Reddi Subhadara, Devaraj Halagowder, Niranjali Devaraj Sivasitambaram

**Affiliations:** 1 Department of Biochemistry, School of Life Sciences, University of Madras, Guindy Campus, Chennai, Tamilnadu, India; 2 Department of Zoology, School of Life Sciences, University of Madras, Guindy Campus, Chennai, Tamilnadu, India; 3 Centre for Biotechnology, Anna University, Chennai, Tamilnadu, India; University of Barcelona, Spain

## Abstract

Plumbagin, a quinonoid found in the plants of the Plumbaginaceae, possesses medicinal properties. In this study we investigated the anti-proliferative and apoptotic activity of plumbagin by using two human colonic cancer cell lines, HT29 and HCT15. IC50 of Plumbagin for HCT15 and HT29 cells (22.5 µM and 62.5 µM, respectively) were significantly different. To study the response of cancer cells during treatment strategies, cells were treated with two different concentrations, 15 µM, 30 µM for HCT15 and 50 µM, 75 µM for HT29 cells. Though activation of NFκB, Caspases-3, elevated levels of *TNF-α*, cytosolic Cytochrome C were seen in both HCT15 cells HT29 treated with plumbagin, aberrant apoptosis with decreased level of pEGFR, pAkt, pGsk-3β, PCNA and Cyclin D1was observed only in 15 µM and 30 µM plumbagin treated HCT15 and 75 µM plumbagin treated HT29 cells. This suggests that plumbagin induces apoptosis in both HCT15 cells and HT29 treated, whereas, proliferation was inhibited only in 15 µM and 30 µM plumbagin treated HCT15 and 75 µM plumbagin treated HT29 cells, but not in 50 µM plumbagin treated HT29 cells. Expression of COX-2 was decreased in 75 µM plumbagin treated HT29 cells when compared to 50 µM plumbagin treated HT29 cells, whereas HCT15 cells lack COX. Hence the observed resistance to induction of apoptosis in 50 µM plumbagin treated HT29 cells are attributed to the expression of COX-2. In conclusion, plumbagin induces apoptosis in colonic cancer cells through TNF-α mediated pathway depending on expression of COX-2 expression.

## Introduction

Colorectal cancer (CRC) is a primary public health concern to the humankind and is the third most common cancer in the United States [1]. Recent data on colorectal cancer is alarming with an estimated 153,760 cases of CRC including 52,180 deaths in 2007 [1], [2]. Progression from normal colonic epithelial cells into a colorectal carcinoma is a multistep process. Even though there many factors such as, loss or mutation in tumor suppressor genes, epigenetic alterations controlling cell survival, cell proliferation and angiogenesis, inflammation play a crucial role in CRC development and progression [3]–[8].

NF-κB is a key inflammatory mediator involved in initiation, progression and metastasis of CRC [9]. A variety of carcinogens and tumor promoters have been shown to activate NF-κB and constitutive expression of NF-κB is frequently found in tumor cells. Several genes involved in tumor initiation, promotion, and metastasis are regulated by NF-κB as well as activation of NF-κB suppresses apoptosis and promotes proliferation. Hence, agents that can down-regulate the activation of NF-κB therefore, would have the potential to inhibit development of cancer.

Plumbagin (5-hydroxy-2-methyl-1,4-naphthoquinone), a quinonoid, found in the plants of the Plumbaginaceae, Droseraceae, Ancestrocladaceae, and Dioncophyllaceae families. The Chief source of Plumbagin is the root of *Plumbago zeylanica* L. (also known as “Chitrak”). Plumbagin has been shown to exert anticarcinogenic, antiatherosclerotic and antimicrobial effects [10]–[13]. The root of *P. zeylanica* L. has been used in Indian medicine for ∼2,750 years and its component possess antiatherogenic, cardiotonic, hepatoprotective and neuroprotective properties [11].

Sugie et al. [14] have shown that plumbagin significantly inhibited azoxymethane-induced intestinal carcinogenesis in rats, suggesting its chemopreventive activity. Plumbagin has also been shown to induce S-G2/M cell cycle arrest through the induction of p21 (an inhibitor of cyclin-dependent kinase) [15]. Recent studies showed that Plumbagin induces apoptosis through inhibition of NF-κB in various cancer cell lines including human chronic myeloid leukemia, human multiple myeloma, human embryonic kidney carcinoma and Breast cancer cells [16], [17]. In this study, we examined the anti-proliferative and apoptotic activity of plumbagin by *in vitro* experimental models using two human colonic cancer cell lines, HT29 and HCT15. Further, the mechanism of anticancer activity of plumbagin was established by analysing cell cycle regulation and signalling molecules related to cell survival and apoptosis.

## Materials and Methods

### Cell culture and maintenance

Human colon tumor cell lines HT29 and HCT15 were obtained from NCCS Pune. Cells were grown in Dulbeccos Modified Eagle Medium (DMEM, GIBCO BRL, Germany) supplemented with 10% FBS (Sigma, USA), 100 units/ml Penicillin, 100 µg/ml Streptomycin, 10–20 µg/ml fungisone (Himedia, India), pH 7.4 in 25 cm^2^, tissue culture flasks (Himedia, India) at 37°C under 5% CO_2_ and 95% air.

### Treatment

Plumbagin was purchased from Sigma. A 100 mM solution of plumbagin was prepared in dimethyl sulfoxide (DMSO), stored as small aliquots at −20°C and then diluted as needed in cell culture medium. Dose-response studies were carried out to determine the suitable dose for the inhibition of cell growth and induction of apoptosis.

### MTT assay

Sensitivity of HT29 and HCT15 cells to plumbagin was determined by the MTT colorimetric assay [18]. Cells (1×10^3^ per well) were seeded in a flat-bottomed 96-well plate and incubated for 24 h at 37°C and in 5% CO2. Both cell lines were exposed to plumbagin (1, 2.5, and 5 µM for 24 h). The solvent DMSO treated cells served as control. Cells were then treated with MTT reagent (10 µl/well) for 4 h at 37°C and then isopropanol (100 µl) was added to each well to dissolve the formazan crystals. The optical density (OD) was recorded at 570 nm in a microplate reader. Percentage of residual cell viability was determined as [1−(OD of treated cells/OD of control cells)]×100.

### Effect of plumbagin on PBMC

PBMC was isolated from heparinized venous blood obtained from a healthy human volunteer by Ficoll-Paque (Histopaque 1077, Sigma Aldrich Inc., USA) density gradient centrifugation as per standard procedure [19]. PBMC (1×105 cells/well) were cultured in complete RPMI-1640 media as usual and incubated with plumbagin for 48 h followed by MTT assay.

### Cell cycle analysis using flow cytometry

Both HT29 and HCT15 cells were seeded in a 6-well plate at a density of 1×10^5^ cells per well. Cells were trypsinized and harvested by centrifugation at 1700 *g*. Flow cytometric analysis of cell cycle was performed by staining of permeabilized cells with propidium iodide (PI) for DNA content. Cells were resuspended in PBS, fixed with 70% ethanol, stained with PI solution (0.05 mg/ml PI, 2 mg/ml RNase A, 0.1% TritonX-100 in PBS) and incubated for 30 min at room temperature (RT) in darkness. Fluorescence intensity was measured by flow cytometry (Becton-Dickinso) using excitation and emission wavelengths of 488 and 525 nm, respectively. All experiments were performed in triplicates.

### Comet assay

The comet assay was performed according to the method of Singh et al. (1988) [20] with minor modifications. HT29 and HCT15 cells (5×10^5^) were seeded into 24-well plates and exposed to the desired concentrations of plumbagin (the final concentration of DMSO did not exceed 0.1%, controls were simultaneously treated with 0.1% DMSO) for specified time periods. Following treatment, cells were pelleted by centrifugation at 1500 rpm. The pellet was resuspended in 60 µl of PBS (pH 7.4). 10 µl of cell suspension was mixed with 100 µl of 1% low melting agarose and 75 µl of this cell–agarose mixture was spread on microscopic slides precoated with 1% agarose. A third layer of 0.5% low melting agarose (75 µl) was applied over the layer of agarose with the cell suspension. Slides were incubated for 1 h in a lysis solution (2.5 M NaCl, 100 mM Na2EDTA, 10 mM Tris, 1% Triton X- 100, 1% SDS, pH 10). Subsequently cells were exposed to an alkaline buffer (300 mM NaOH, 1 mM disodium EDTA) for 30 min. The microscopic slides were subjected to electrophoresis at 0.8 V/cm−1 for 30 min after which slides were immersed in a neutralization buffer (0.4 M Tris, pH 7.5) for 15 min. After staining with ethidium bromide (20 µg/ml) slides were analyzed under a fluorescence microscope (Nikon PCM-2000). Images of at least 50 cells from three slides were analyzed using Cometscore™ software. For each comet two areas were selected: the whole cellular DNA and an area containing only the head region of the comet. In each selection the densities were measured and the results were presented as tail moment defined as the result of the percentage of DNA in the tail multiplied by the tail length.

### Isolation of RNA

For preparation of total RNA, the phenol – guanidinium thiocyanate based Tri Reagent (GeNei™, Bangalore) was used. To 10^7^ cells, 1 ml of Tri reagent was added and lysed by repetitive pipetting and allowed to stand for 5 min followed by addition of 200 µl of chloroform for phase separation .Vigorously vortexed for 15 sec and allowed to stand for 15 min followed by centrifugation at 12000 g for 15 min at 4°C. The upper aqueous layer containing RNA was transferred to a fresh sterile DEPC treated microfuge tube. To this, 500 µl of ice cold isopropanol was added, gently mixed and allowed to stand for 10 min and centrifuged at 12000 g for 15 min at 4°C. The supernatant was discarded and the RNA pellet was washed with 1 ml of 75% ethanol in DEPC treated water and again centrifuged at 14000 g for 10 min at 4°C to get total RNA. This pellet was dissolved in 25 µl of sterile RNase free water by heating at 55°C for 20 min and stored at −20°C until use.

### Reverse transcriptase polymerase chain reaction (RT-PCR)

To synthesize cDNA, a reverse transcription reaction solution containing 1.0 µg total RNA in RNase/DNase-free water and 1.5 µl of random hexamer primer (GeNeiTM, Bangalore) were incubated for 10 min at 72°C and chilled immediately. To this, 5.0 µl premixed 10 mM dNTP solution (GeNeiTM, Bangalore), 3.0 µl 10× M-MLV reverse transcriptase buffer (GeNeiTM, Bangalore), 1.0 µl (200 units/µl) M-MLV reverse transcriptase (GeNeiTM, Bangalore), were added and made up to 50 µl using sterile RNase/DNase-free water.

To amplify the cDNA, polymerase chain reaction (PCR) ready mix (GeNeiTM, Bangalore) was used according to manufacturer's instruction. All PCR samples were denatured at 94°C for 5 min prior to cycling and were extended for 10 min at 72°C following cycling. The PCR assay using primers was performed for 39 cycles at 94°C for 60 s, 60°C for 60 s, and 72°C for 60 s. Primers for GAPDH, COX-2, TNF-α are shown in Table 1. Primers were designed using primer3 software available free on http://fokker.wi.mit.edu/primer3/input.htm and nucleic acid sequence was accessed from http://www.ncbi.nlm.nih.gov/entrez/viewer.fci?val=Reference ID Table. The primers were purchased from Integrated DNA technologies, USA. Further, expression of COX-2 and TNF-α was analysed semi quantitatively using UN SCAN IT software.

**Table 1:** Primer sequence of GAPDH, COX-2 and TNF-α.

S.No.	Gene	Primer pair	Product size (bp)
1.	*GAPDH*	Left 5′- ACAGTCAGCCGCATCTTCTT -3′	312
		Right 5′- TTGATTTTGGAGGGATCTCG -3′	
2.	*COX-2*	Left - TGAGCATCTACGGTTTGCTG -3′	158
		Right 5′- TGCTTGTCTGGAACAACTGC -3′	
3	*TNF-α*	Left - CTATCTGGGAGGGGTCTTCC -3′	134
		Right 5′- ATGTTCGTCCTCCTCACAGG -3′	

### Isolation of protein

To 0.5 ml phenol-ethanol supernatant, 3 volumes of acetone were added to precipitate proteins. Samples were mixed by inversion for 10–15 sec to obtain a homogeneous solution and allowed to stand for 10 min at room temperature. Protein was precipitated by centrifuging at 12,000 g for 10 min at 4°C. The supernatant was removed and pellet dispersed in 0.5 ml of 0.3 M guanidine hydrochloride in 95% ethanol with 2.5% glycerol (V∶V). After dispersing the pellet, another 0.5 ml aliquot of the guanidine hydrochloride/ethanol/glycerol wash solution was added to the sample and stored for 10 min at room temperature. Protein was pelleted at 8,000 g for 5 min at 4°C. The wash solution was removed and two more washes were performed in 1 ml each of the guanidine/ethanol/glycerol wash solution. Final wash was performed with 1 ml of ethanol containing 2.5% glycerol (V∶V). Protein was pelleted out by centrifuging at 8000 g for 10 min at 4°C and air dried for 5 min. After briefly air-drying, the protein pellet was dissolved in 1% SDS. Protein concentrations of cell lysates were estimated according to Lowry et al., 1951 [21] and equal concentrations of protein fraction were run on sodium dodecyl sulfate-polyacrylamide gel electrophoresis (SDS-PAGE) and immunobloted with specific antibodies.

### siRNA transfection

COX-2 gene was silenced by transfecting COX-2 siRNA. In a six well tissue culture plate, 2×10^5^ cells per well were seeded in 2 ml antibiotic-free normal growth medium supplemented with FBS. Cells were incubated at 37°C in a CO_2_ incubator until the cells were 60–80% confluent. Cell viability was assessed before transfection. The following solutions were prepared: Solution A: For each transfection, diluted 6 µl of siRNA duplex (i.e., 0.25–1 µg or 20–80 pmols siRNA) into 100 µl siRNA Transfection Medium (Santa cruz, USA). Solution B: For each transfection, dilute 6 µl of siRNA Transfection Reagent (Santa cruz, USA) into 100 µl siRNA Transfection Medium. siRNA duplex solution (Solution A) was mixed with diluted Transfection Reagent (Solution B) and incubate the mixture 45 minutes at room temperature. For each transfection, 0.8 ml siRNA Transfection Medium was added to each tube containing the siRNA Transfection Reagent mixture (Solution A + Solution B), mixed gently, overlaid onto washed cells and Incubated for 5–7 hours at 37°C in a CO_2_ incubator. After incubation, 1 ml of normal growth medium containing 2 times the normal serum and antibiotics concentration (2× normal growth medium) were added without removing the transfection mixture. Cells were incubated for an additional 18–24 hours. Medium was replaced with 1 ml of fresh 1× normal growth medium and used for further assay.

### Statistical analysis

Values were recorded as the mean x ± SD of three experiments. Experimental results were analyzed by student's t-test. P<0.00 was considered as the level of highly significance and P<0.05 was considered as significance for values obtained for treated groups compared with control group.

## Results

### Plumbagin induces cytotoxicity in HCT15 and HT29 colon cancer cells

**Figure 1:**
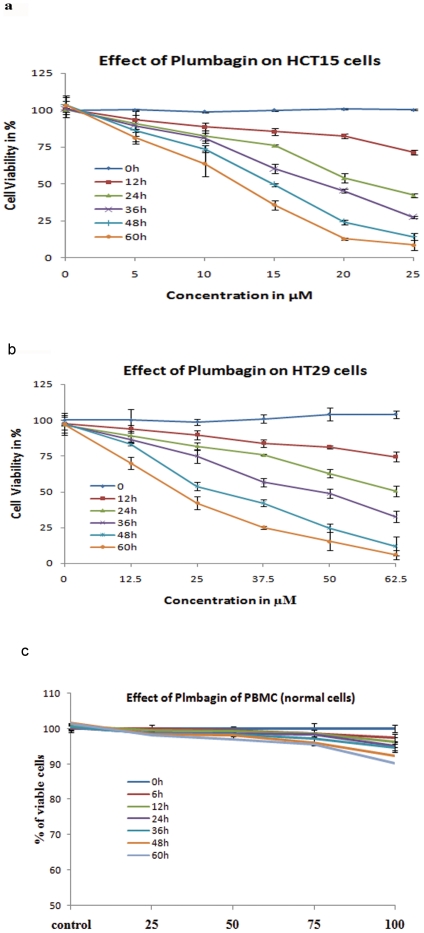
Effect of Plumbagin on Colonic cancer cells and normal PBMCs. *a. Cytotoxicity effect of various concentrations of Plumbagin on HCT15 cells*. Dose dependent cytotoxicity effects of Plumbagin on HCT15 cells were represented in the above graph. HCT15 cells were more sensitive to plumbagin as IC_50_ at 24 hours was 22.5 µM. All the experiments were done in triplicates and expressed as the mean ± SD. Significance is indicated as *p<0.001. *b. Cytotoxicity effect of various concentrations of Plumbagin on HT29 cells*. Dose dependent cytotoxicity effects of Plumbagin on HT29 cells were represented in the above graph. HT29 cells were more sensitive to plumbagin as IC_50_ at 24 hours was 62.5 µM. All the experiments were done in triplicates and expressed as the mean ± SD. Significance is indicated as *p<0.001. *c. Cytotoxicity effect of various concentrations of Plumbagin on PBMC cells*. Dose dependent cytotoxicity effects of Plumbagin on PBMC cells were represented in the above graph. PBMC cells were resistance to plumbagin induced cytotoxicity even at 100 µM concentration. All the experiments were done in triplicates and expressed as the mean ± SD. Significance is indicated as *p<0.001.

Employing HCT15 and HT29 colon cancer cell lines, we first evaluated the effect of cytotoxicity of plumbagin by MTT assay. In addition to aberrant Wnt signaling in both cell lines, HT29 cells are known to express COX2, whereas HCT15 cells lack COX2 expression. Both cell lines were sensitive to plumbagin, indicating that plumbagin could induce cytotoxicity of both the colon cancer cells regardless of COX2 status (**Fig. 1a and 1b**). However, HCT15 cells were more sensitive to plumbagin as IC_50_ at 24 hours was 22.5 µM, which is significantly lower than that of IC_50_ of plumbagin treated HT29, which is 62.5 µM. Only non significant changes in viability of PBMC's (normal cells) were observed even in100 µM of Plumbagin incubated cells (**Fig. 1c**)

### Plumbagin inhibits proliferation of HCT15 and HT29 colonic cancer cells

**Figure 2:**
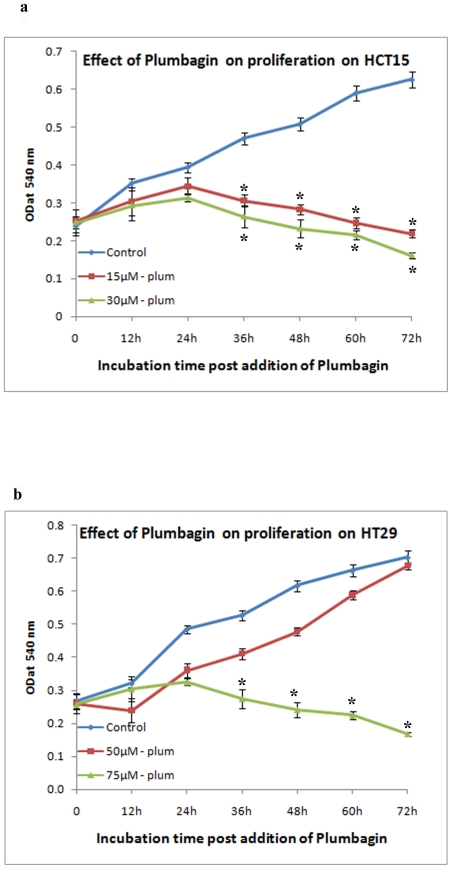
Effect of different concentrations of Plumbagin on HCT15 and HT29 cell proliferation. *a. HCT15 cells. b. HT29 cells*. Proliferation of HCT15 cells treated with 15 µM and 30 µM of plumbagin and HT29 cells treated with 75 µM plumbagin increased significantly at 48 h and 72 h, whereas , 50 µM plumbagin treated HT29 cells tend to proliferate. All the experiments were done in triplicates and expressed as the mean ± SD. Significance is indicated as *p<0.001.

The proliferation of HCT15 cells treated with 15 µM and 30 µM of plumbagin increased slightly at 24 h and decreased significantly at 48 h and 72 h, whereas untreated control cells were maintained exponential growth phase. In contrast, HT29 cells treated with 50 µM plumbagin showed exponential growth, similar to that of untreated control cells, whereas, the growth of HT29 cells treated with 75 µM plumbagin increased slightly at 24 h and decreased significantly at 48 h and 72 h (**Fig. 2a and 2b**).

### Plumbagin induces apoptosis in HCT15 and HT29 colon cancer cells

**Figure 3:**
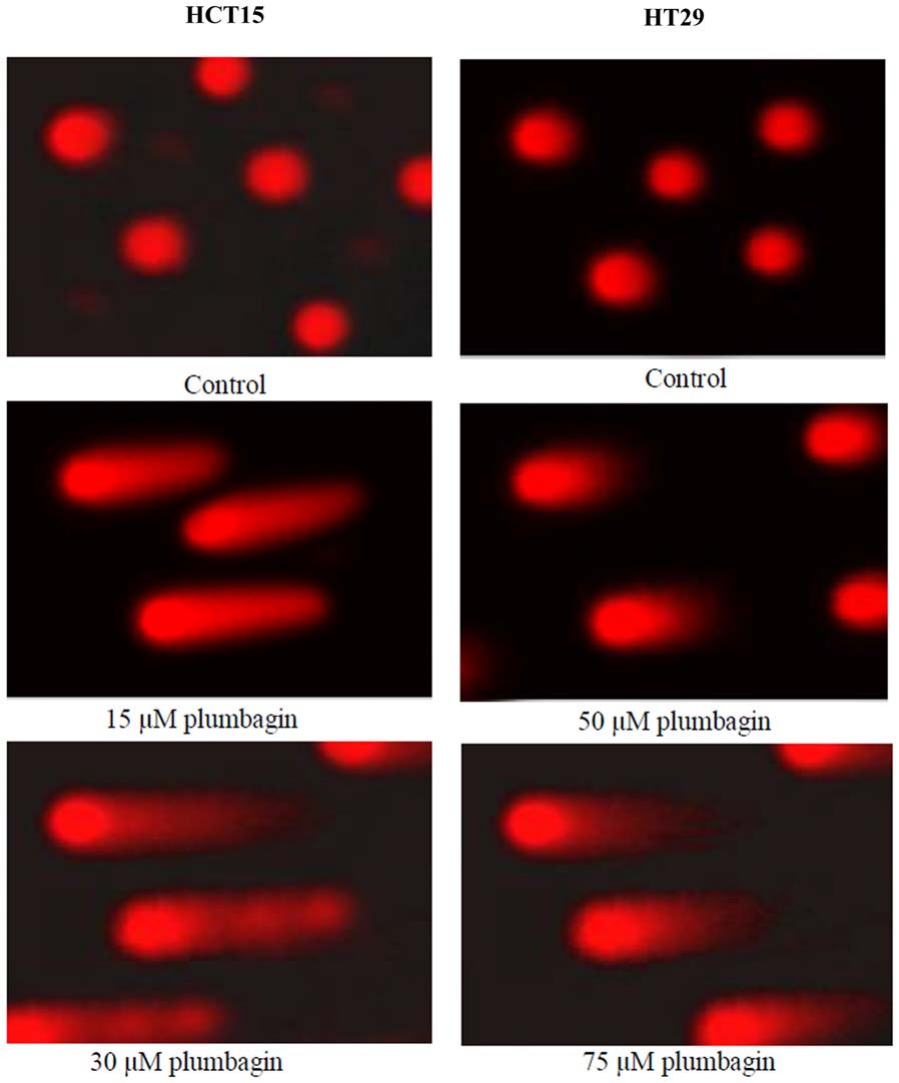
Analysis of apoptotic inducing effect of plumbagin on HCT15 and HT29 cells assessed by comet assay. HCT15 cells treated with 30 µM plumbagin and HT29 cells treated with 75 µM plumbagin showed increased extant of DNA damage. The length of the comet tail was ten and six times that of the control and 15 µM plumbagin treated HCT15, respectively, while, 50 µM and 75 µM plumbagin - treated HT29 cells shows four and two time of span of tail, respectively, compared to control HT29 cells. DNA damage was not observed, as the halo surrounding cell nuclei was clearly visible in control cells.

To determine the DNA damaging properties of plumbagin, HCT15 cells were incubated with 15 µM and 30 µM plumbagin for 24 h, whereas, HT29 cells were incubated with 50 µM and 75 µM plumbagin for 24 h. The appearance of comet tails corresponding with the induction of DNA damage were visible (**Fig. 3**). HCT15 cells treated with 30 µM plumbagin and HT29 cells treated with 75 µM plumbagin showed increased extant of DNA damage. The span of the comet tail was ten and six times that of the control HCT15 cells and 15 µM plumbagin treated HCT15, respectively, while, 50 µM and 75 µM plumbagin - treated HT29 cells shows four and two time of span of tail, respectively, compared to control HT29 cells. DNA damage was not observed, as the halo surrounding cell nuclei was clearly visible in control cells.

### Analysis of NFκB, Caspase-3 activation and cytochrome C release in plumbagin incubated HCT15 and HT29 cells

**Figure 4:**
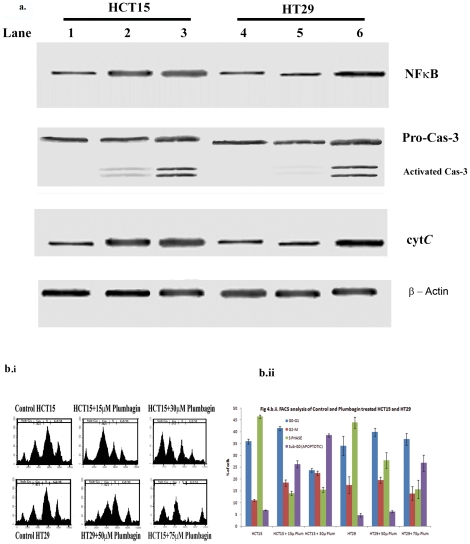
Plumbagin induces apoptotic in colonic cancer epithelial cells. *a. Immunoblotting analysis of NFκB activation, Caspase-3 activation and cytochrome C release*. Lane 1- Control HCT15; Lane 2- 15 µM plumbagin treated HCT15; Lane 3- 30 µM plumbagin treated HCT15; Lane 4- Control HT29; Lane 5- 50 µM plumbagin treated HT29; Lane 6- 75 µM plumbagin treated HT29. *b.i. Cell cycle analysis of control and plumbagin treatment HCT15 and HT29 cells by Flow Cytometry*. Compared with control HCT15, HCT15 treated with plumbagin shows marked rise in sub-G1 fraction suggesting that these cells are undergoing apoptosis. HT29 cells exposed to 75 µM of Plumbagin alone exhibited increase in sub-G1 fraction whereas HT29 cells exposed to 50 µM of Plumbagin sub-G1 fraction was much lesser. *b.ii. Quantitative data of cell cycle analysis*. All the experiments were done in triplicates and expressed as the mean ± SD. Significance is indicated as *p<0.001.

Activation of Caspase-3, NFκB (p65) and cytosolic release of cytochrome C were analysed by western blotting (**Fig. 4a**). Activation of NFκB (p65) was significantly increased in both 15 µM and 30 µM plumbagin - treated HCT15 as well as in 50 µM and 75 µM plumbagin - treated HT29 cells, when compared to control HCT15 and HT29 cells. Activation of Caspase-3 and levels of cytosolic cytochrome C were significantly high, in both 15 µM and 30 µM plumbagin - treated HCT15 cells, when compared to untreated HCT15 cells. However activation of Caspase-3 and levels of cytosolic cytochrome C were high only in 75 µM plumbagin treated HT29 cells, but not in HT29 cells treated with 50 µM plumbagin.

### Cell cycle analysis of control and plumbagin treated HCT15 and HT29 cells

HCT15 cells exposed to 15 and 30 µM of Plumbagin exhibited continuous increase in sub-G1 fraction which may include both apoptotic and debris fraction implying together the extent of cell death. The damage was more apparent with 30 µM of Plumbagin. The sub-G1 fraction for control was 6.8% whereas the same was 24.52 and 37.87% for 15 and 30 µM of Plumbagin treated HCT15 cells respectively (**Fig. 4b.i**). This indicates a dose dependant increase in apoptosis on HCT15 cells by Plumbagin. Results also illustrated associated accumulation of cells in G0/G1 phase, indicating inhibition of movement of cells from G0/G1 phase to S phase, thus delaying or inhibiting the entry of daughter cells into mitotic cycle.

In case of HT29 cells exposed to 75 µM of Plumbagin alone exhibited increase in sub-G1 fraction whereas HT29 cells exposed to 50 µM of Plumbagin sub-G1 fraction was much lesser. The sub-G1 fraction for control was 4.39% whereas the same was 6.14 and 26.76% for 50 and 75 µM of Plumbagin treated H29 cells respectively, indicating occurrence of extensive apoptosis only in 75 µM of Plumbagin treated H29 cells, when compared to 50 µM of Plumbagin treated H29 cells and control HT29 cells. Results also illustrate that accumulation of cells in G0/G1 phase only in 75 µM of Plumbagin treated H29 cells, causing significant decrease in population of S and G2/M phase, which indicates delay or inhibition of entry of these cells into synthesis phase and mitotic cycle. Significantly increased cell population were seen in S and G2/M phase in 50 µM of Plumbagin treated H29 cells indicating proliferation of cells (**Fig. 4.b.ii**)

### Analysis of phosphorylated Akt, phosphorylated EGFR, PCNA and cyclin D1 in plumbagin incubated HCT15 and HT29 cells

**Figure 5:**
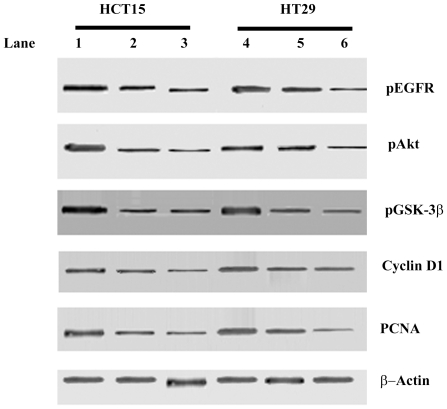
Western blotting analysis of phosphorylated Akt, phosphorylated EGFR, PCNA and cyclin D1 in control and plumbagin treated HCT15 and HT29 cells. Lane 1- Control HCT15; Lane 2- 15 µM plumbagin treated HCT15; Lane 3- 30 µM plumbagin treated HCT15; Lane 4- Control HT29; Lane 5- 50 µM plumbagin treated HT29; Lane 6- 75 µM plumbagin treated HT29. Expression of PCNA, cyclin D1 along with phosphorylation of Akt and EGFR were significantly decreased in 15 µM and 30 µM plumbagin treated HCT15 and in 75 µM plumbagin when compared to Control HCT15, Control HT29 and HT29 cells treated with 75 µM plumbagin. β-Actin served as internal control.

Expression of PCNA, cyclin D1 along with phosphorylation of Akt and EGFR was analysed by western blotting (**Fig. 5**). Expression of PCNA was significantly decreased in 15 µM and 30 µM plumbagin treated - HCT15 cells when compared with untreated cells. Significant decrease in levels of PCNA was observed only in 75 µM plumbagin treated HT29 cells but not in 50 µM plumbagin treated HT29 cells. Significantly decreased levels of phosphorylated EGFR, Akt and Gsk-3β were observed in 15 µM and 30 µM plumbagin treated HCT15 and in 75 µM plumbagin treated HT29 cells when compared to control cells. In 50 µM plumbagin treated HT29 cells, levels of phosphorylated EGFR, Akt and Gsk-3β showed insignificant changes. β - Actin served as internal control.

### Analysis of *TNF-α* and *cox-2* expression in HCT15 and HT29 cells treated with plumbagin

**Figure 6:**
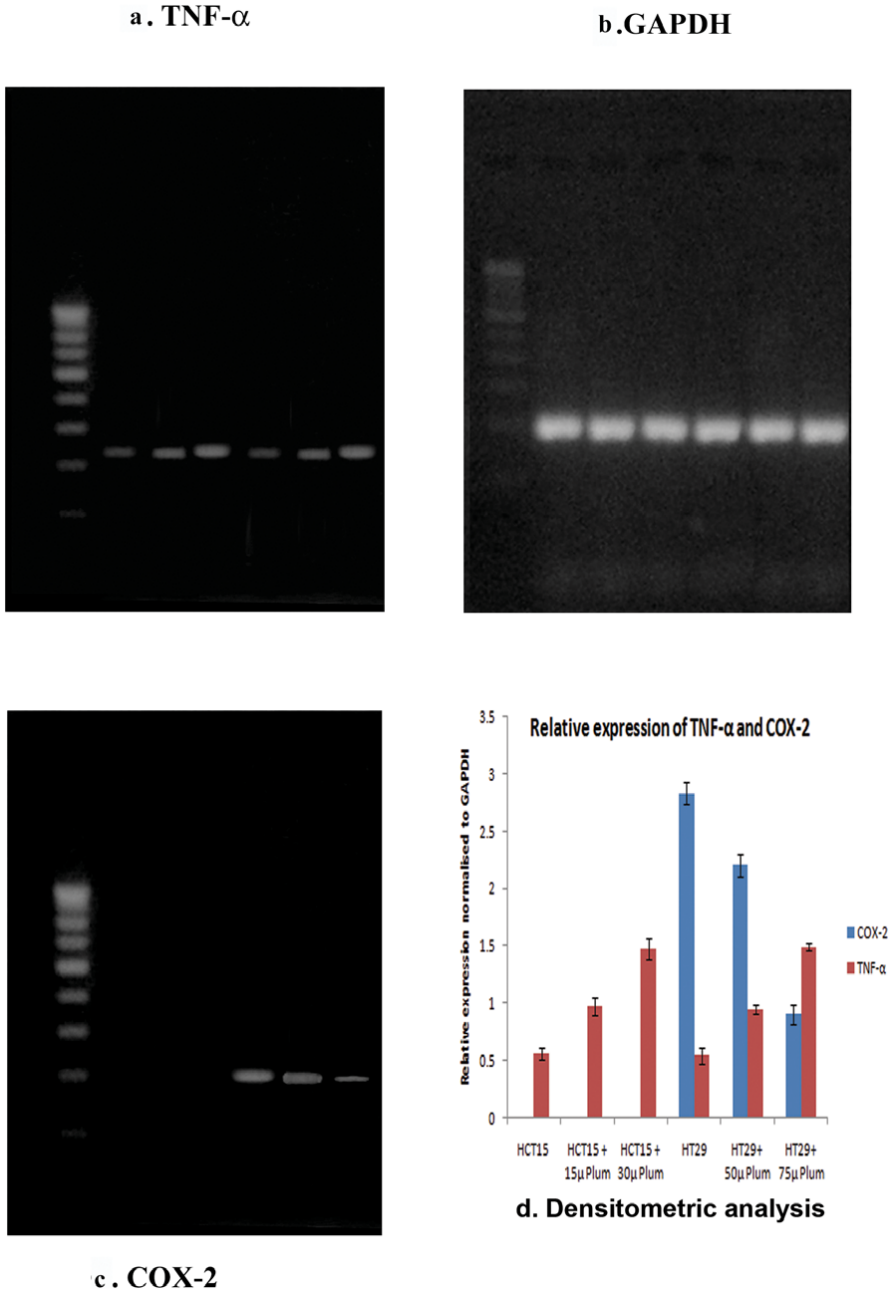
Expression analysis of TNF-α, COX-2 and GAPDH by RT-PCR. Lane 1- Control HCT15; Lane 2- 15 µM plumbagin treated HCT15; Lane 3- 30 µM plumbagin treated HCT15; Lane 4- Control HT29; Lane 5- 50 µM plumbagin treated HT29; Lane 6- 75 µM plumbagin treated HT29. a. Plumbagin - treated HCT15 and HT29 cells showed increased amounts of RNA transcripts of the *TNF-α* gene compared to the untreated HCT15 and HT29 cells. b. Levels of RNA transcript of *cox-2* was decreased in 75 µM plumbagin treated HT29 cells, when compared to 50 µM plumbagin treated HT29 cells, while plumbagin treated HCT15 cells as well as untreated HCT15 did not show any expression of *cox-2* RNA transcript. c. Showing level of GAPDH RNA transcripts in control and Plumbagin - treated HCT15 and HT29 cells. d. Represents densitometric analysis showing relative expression of *TNF-α* and *cox-2* to GAPDH. Represented data values were obtained from triplicate analysis and expressed as the mean ± SD. Significance is indicated as *p<0.05; **p<0.001.

Both, 15 µM and 30 µM plumbagin - treated HCT15 and 50 µM and 75 µM plumbagin - treated HT29 cells showed increased amounts of RNA transcripts of the *TNF-α* gene compared to the untreated HCT15 and HT29 cells (**Fig. 6a**). Levels of RNA transcript of *cox-2* was decreased in 75 µM plumbagin treated HT29 cells, when compared to 50 µM plumbagin treated HT29 cells, while plumbagin treated HCT15 cells as well as untreated HCT15 did not show any expression of *cox-2* RNA transcript (**Fig. 6c**). *GAPDH* was used as internal control (**Fig. 6b**). Comparing control HCT15 and HT29 cells, increased relative expression of *TNF-α* was seen in both HCT15 and HT29 cells treated with Plumbagin at both concentrations while relative expression of *cox-2* is significantly decreased only in 75 µM plumbagin treated HT29 cells, when compared to 50 µM plumbagin treated HT29 cells (**Fig. 6d**). Represented data values were obtained from triplicate analysis and expressed as the mean ± SD. Significance is indicated as *p<0.05; **p<0.001.

### Role of COX-2 in resistance to plumbagin induced apoptosis in HT29 colon cancer cells

**Figure 7:**
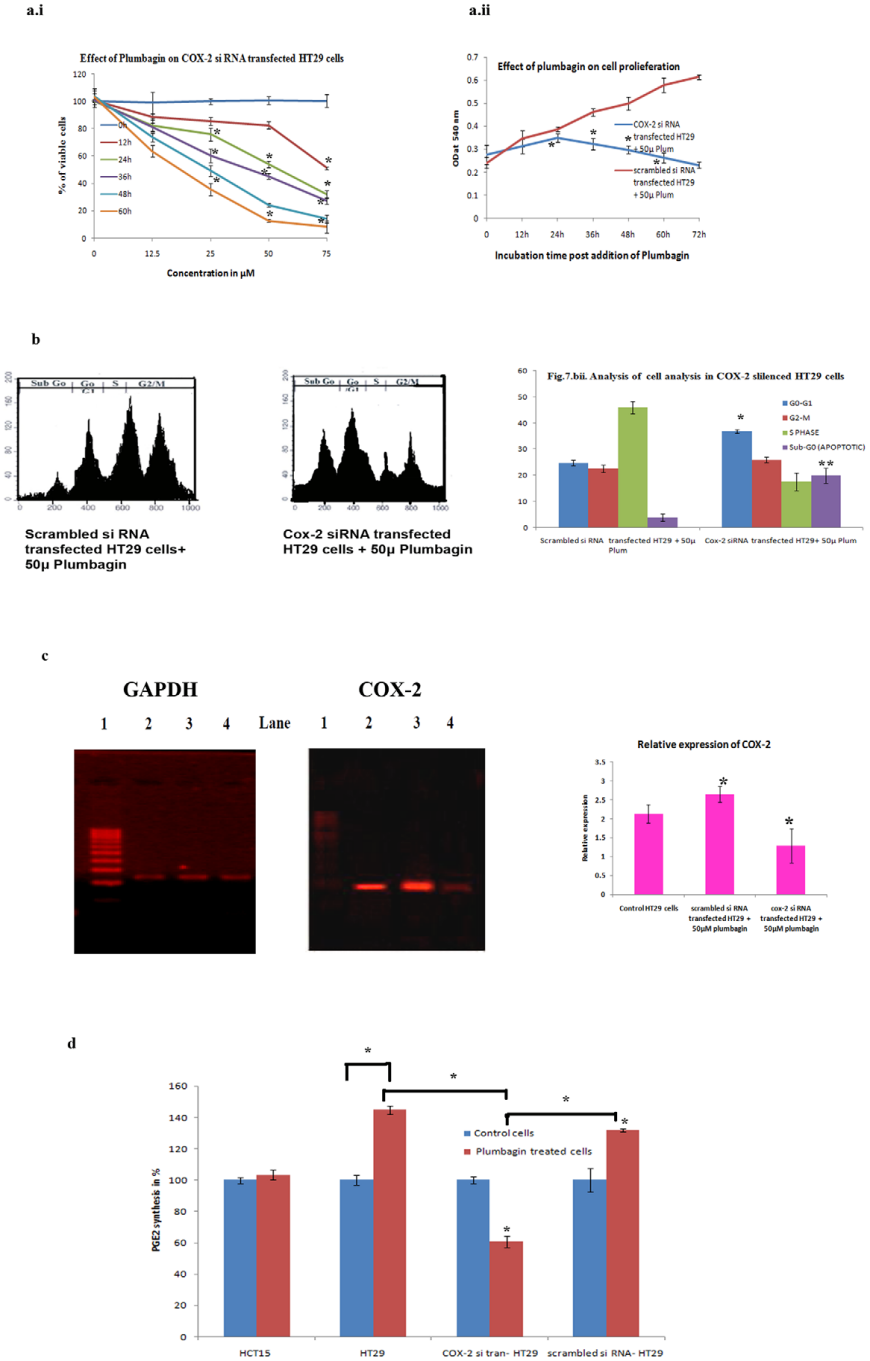
Role of COX-2 in resistance to Plumbagin induced apoptosis. *a.i. Cytotoxicity effect of various concentrations of Plumbagin on COX-2 silenced HT29 cells*. Dose dependent cytotoxicity effects of Plumbagin on HCT15 cells were represented in the above graph. HCT15 cells were more sensitive to plumbagin as IC_50_ at 24 hours was 22.5 µM. All the experiments were done in triplicates and expressed as the mean ± SD. Significance is indicated as *p<0.001. *a.ii. Effect of 50 µM Plumbagin on proliferation of COX-2 silenced HT29 cells and scrambled siRNA transfected HT29 cells*. Proliferation of COX-2 silenced HT29 cells was significantly inhibited by 50 µM plumbagin treatment, whereas, 50 µM plumbagin treated scrambled siRNA transfected HT29 cells tend to proliferate. All the experiments were done in triplicates and expressed as the mean ± SD. Significance is indicated as *p<0.001. *b. Cell cycle analysis in 50 µM Plumbagin treated COX-2 silenced HT29 cells and scrambled siRNA transfected HT29 cells*. Compared to plumbagin treated scrambled siRNA transfected HT29 cells, plumbagin treated COX-2 silenced HT29 cells shows marked rise in sub-G1 fraction suggesting that these cells are undergoing extensive apoptosis. Quantitative also clearly supports this fact. All the experiments were done in triplicates and expressed as the mean ± SD. Significance is indicated as *p<0.001. *c. COX-2 Expression analysis in 50 µM Plumbagin treated COX-2 silenced HT29 cells and scrambled siRNA transfected HT29 cells*. Lane 1 – Marker, Lane 2 – Control cells, Lane 3 – scrambled si RNA transfected HT29+50 µM plumbagin, Lane 4 – cox-2 si RNA transfected HT29+50 µM plumbagin. Represents densitometric analysis showing relative expression of *cox-2*. Represented data values were obtained from triplicate analysis and expressed as the mean ± SD. Significance is indicated as *p<0.001. *d. Analysis of PGE2 levels*. All the experiments were done in triplicates and expressed as the mean ± SD. Significance is indicated as *p<0.001.

To determine the role of COX-2 in resistance of Plumbagin induced apoptosis in HT29 colon cancer cells, COX-2 was silenced and cytotoxicity analysis and cell proliferation was done. Plumbagin of significantly induced cytotoxicity in COX-2 silenced HT29 cells in dose dependent manner, where IC_50_ at 24 hours was found to be 55.5 µM (**Fig. 7.a.i**). It also significantly inhibited cell proliferation in COX-2 silenced HT29 cells compared to scrambled siRNA transfected HT29 cell incubated with 50 µM of Plumbagin (**Fig. 7.a.ii**).

Further, cell cycle analysis was assessed using florescent activated cell sorting analysis (FACS). COX-2 siRNA transfected HT29 cells treated with 50 µM of plumbagin showed 36.78% of cells in G_0_G_1_ phase while 17.64% and 25.88% cells were in S phase and G_2_M phase, respectively. 19.86% of plumbagin treated HT29 cells were undergoing apoptosis (**Fig. 7.b.i & Fig. 7.b.ii**), while in scrambled cox-2 siRNA transfected untreated HT29 cells showed 24.36% of cells were in G_0_G_1_ phase while 45.93% and 22.54% of cells were in S phase and G_2_M, respectively. 3.8% of control cells were undergoing apoptosis (**Fig. 7.b.i & Fig. 7.b.ii**).

Relative expression of COX-2 in Plumbagin treated COX-2 si RNA transfected HT29 cells are significantly (P<0.001) decreased when compared to scrambled si RNA transfected HT29 cells treated with Plumbagin (**Fig. 7.c**).

Synthesis of prostaglandinE2 (PGE_2_) was unchanged in Plumbagin treated HCT15 as compared to that of the control HT15 cells. Significantly (P<0.001) decreased level of PGE_2_ was observed in Plumbagin treated COX-2 si RNA transfected HT29 cells when compared with Plumbagin treated HT29 cells and scrambled si RNA transfected HT29 cells treated with Plumbagin (**Fig. 7.d**).

## Discussion

Colorectal cancer is the most common neoplasm in human in both developed and developing countries. Many of chemotherapeutic agent used is cancer treatment have side effects. On the other hand, the use of nonsteroidal anti-inflammatory drugs exerts antitumor effects against CRC [8], however, inflammation favours proliferation as well as angiogenesis that support the role of inflammatory mechanisms in growth and progression of CRC.

Traditional medicines, although argued to be harmless and effective, in most cases neither the chemical entity nor the molecular mechanisms of action were well defined. *Plumbago zeylanica* is once such medicinal plant used traditionally as an anti inflammatory agent whose mechanism of action has to be established, particularly in cancer. Plumbagin is an active compound of *Plumbago zeylanica*, which has been used as a chemotherapeutic agent for some of cancer including lung carcinoma and mylomas. In our study, we have exemplified the apoptosis inducing ability of plumbagin on two colon cancer cell lines HCT15 and HT29.

In colon cancer, 90% of the tumor occurs as a result of activating mutations in the Wnt pathway [22]. Genetic inclination, such as mutations in adenomatous polyposis coli or β-catenin causes stabilization and activation of β-catenin, which leads to uncontrolled proliferation of intestinal epithelial cells through the constitutively active Wnt-signaling pathway [23], [24]. However, inflammatory mediators such as NFκB, COX and iNOS also play crutial roles in the development of colon cancer and its progression.

Cyclooxygenase (COX), known as prostaglandin (PG) H2 synthase, is the rate-limiting enzyme in the conversion of arachidonic acid into PGs. Overexpression of COX2 has been frequently observed in colon tumors and COX2 plays a major role in colon carcinogenesis [25]. Many studies have revealed that PGE2, the metabolite of COX2, is an effective mitogen, which contributes to the development of colon cancer and targeting COX-2 is one recent therapeutic method for treatment of colon cancer [26], [27]. Hence to assess the vital role played by COX2 in resisting cancer treatment, we employed two colon cancer cell lines, HCT15 and HT29, where, both the cell lines possess aberrant Wnt signalling, but expression of COX-2 was seen only in HT29.

In our study, we demonstrate that in a dose-dependent manner plumbagin induces cytotoxicity effectively in HCT15 cells when compared to the HT29 cells. Accordingly, IC_50_ value of HT29 cells (62.5 µM) was much higher than that of HCT cells (22.5 µM). Hence, further study was performed by treating cells with two different concentrations, one concentration fixed well below the IC_50_ value (i,e 15 µM and 50 µM of plumbagin for HCT15 and HT29, respectively) while the other concentration was fixed above the IC_50_ (i,e 30 µM and 75 µM of plumbagin for HCT15 and HT29, respectively). This has been done to study the response of cancer cells during treatment strategies. However, plumbagin did not exhibit any significant toxicity on normal cells (PBMC) at any of the above concentrations, suggesting that plumbagin possesses selectivity between normal and cancer cells.

Plumbagin alone has been shown to induce apoptosis in different cell types. Our comet assay data also suggest that both the concentrations of Plumbagin induce apoptosis in HCT15. Interestingly, HT29 cells showed resistant to Plumbagin induced apoptosis at 50 µM concentration which is below the IC_50_. However, treatment of 75 µM concentration of Plumbagin induced apoptosis in HT29 cells. Proliferation of HCT15 cells was inhibited at both 15 µM and 30 µM concentration of plumbagin, whereas, Proliferation was inhibited only in 75 µM plumbagin treated HT29, while, 50 µM plumbagin treated HT29 cells tend to proliferate. PCNA data also confirmed the above fact. The above fact is attributed to the observed resistance to induction of apoptosis by Plumbagin in HT29 cells when treated at 50 µM plumbagin.

Previous reports have suggested that plumbagin induce apoptosis through the activation of caspases-3 and Cytochrome-C [16], we examined caspases-3 activation and Cytochrome C release. Activation of caspase-3 and cytosolic Cytochrome C were found to be elevated in 15 µM, 30 µM plumbagin treated HCT15 and 75 µM plumbagin treated HT29 cells. Activation of caspase-3 and release of Cytochrome C by plumbagin are mediated through over expression of TNF-α. Activation of caspase-3 and Cytochrome C were not significantly observed in 50 µM plumbagin - treated HT29 cells, even at elevated levels of TNF-α. This result was interesting, as elevated TNF-α tend to induce apoptosis through TNFR-death domine receptor [28].

Since the only difference between these two cell lines are COX-2 and that could mediate a balance between apoptosis and cell survival, we analysed the expression of COX-2 along with its transcription factor NFκB. Decreased expression of *cox-2* transcripts in 75 µM plumbagin treated HT29 indicates that COX-2 plays a crucial role in plumbagin induced apoptosis. Further, lack of expression of *cox-2* transcripts in control and plumbagin treated HCT15 authenticates the above facts. However, only non significant changes were observed in the level of NFκB transcripts.

As COX-2 is regulated by activated NFκB, we analysed activation NFκB by immuno blotting with p65 specific antibodies. Our results indicate that NFκB activation was found elevated in Plumbagin treated HCT15 and HT29 cells at both the concentrations. Activation of NFκB (p65) is due to elevated levels of TNF-α through TRADD-TRAF. Expression of COX-2 is decreased even at high levels of p65 (activated NFκB) which cloud be due to the fact that plumbagin modulates p65 by targeting Cystine-38. Modification of Cystine-38 in p65 affects its DNA binding capacity [16].

Studies in human cancer indicate that use of specific COX2 inhibitors may be an effective approach for colorectal cancer prevention and treatment [1], [29]. PGE2 is a metabolite of COX-2 and an important downstream target of PGE2 is the epidermal growth factor receptor (EGFR) pathway that has also been implicated in colon carcinogenesis [30]. Hence we analysed phosphorylated EGFR and one of its downstream targets Akt. Increased levels of phosphorylated EGFR and Akt in 50 µM plumbagin treated HT29 cells is contributed by COX-2, thus offering resistance to plumbagin induced apoptosis.

Activation of Akt inhibits Gsk-3β and causing accumulation of cyclin D1 leading to loss of cell cycle regulation [31]–[33]. 15 µM, 30 µM plumbagin treated HCT15 and 75 µM plumbagin treated HT29 cells, cells will be arrested at G_0_/G_1_ phase due to degradation of cyclin D1 by active Gsk-3β as Akt in its inactive form may be due to unavailability of COX-2 dependent PGE2. Akt is still active due to COX-2 dependent phosphorylation of EGFR through PGE2 in 50 µM plumbagin treated HT29 cells showing accumulation of cyclin D1 causing cell cycle disregulation and forms the basis for cell proliferation even with 50 µM plumbagin. Further, increased apoptosis Sub-G1 population and increased cytotoxicity associated with decrease levels of PGE2 in 50 µM Plumbagin treated COX-2 si RNA transfected HT29 cells, authenticate that the COX-2 plays crucial role in resisting Plumbagin induced apoptosis of HT29 cells

Taken into all above facts, we conclude that induction of apoptosis in colonic cancer cells by plumbagin is mediates through TNF-α expression and TNF-α mediated pathway by activating Caspase-3 and releasing of Cytochrome C. However, balance between cell survival and apoptosis controlled by COX-2. Modulation of p65 (NFκB) by Plumbagin inhibits cell survival through inhibiting of phosphorylation of EGFR, Akt and GSK-3β and shifts the balance towards apoptosis.
